# Mild Cognitive Impairment in the Migrant Population Living in Europe: An Epidemiological Estimation of the Phenomenon

**DOI:** 10.3233/JAD-191012

**Published:** 2020-01-21

**Authors:** Marco Canevelli, Valerio Zaccaria, Eleonora Lacorte, Ilaria Cova, Giulia Remoli, Ilaria Bacigalupo, Silvia Cascini, Anna Maria Bargagli, Simone Pomati, Leonardo Pantoni, Nicola Vanacore

**Affiliations:** a National Center for Disease Prevention and Health Promotion, National Institute of Health, Rome, Italy; b Department of Human Neuroscience, “Sapienza” University, Rome, Italy; c Center for Research and Treatment on Cognitive Dysfunctions, “Luigi Sacco” University Hospital, Milan, Italy; d Department of Epidemiology, Regional Health Service, Lazio Region, Rome, Italy; e Department of Biomedical and Clinical Sciences “Luigi Sacco”, University of Milan, Milan, Italy

**Keywords:** Cognitive disorders, health disparities, migration, mild cognitive impairment, neuroepidemiology

## Abstract

**Background:**

The construct of mild cognitive impairment (MCI) is triggering growing clinical and research interest. The detection of MCI may be affected by diverse ethno-cultural determinants possibly influencing the personal and social perception of the individual cognitive functioning as well as the reliability of objective cognitive assessment. These challenges may acquire special relevance in subjects with a migration background and composing ethnic minority groups.

**Objective:**

The present study is aimed at providing an estimate of the number of MCI cases occurring in the migrant population living in the extended European Union (EU) in 2018.

**Methods:**

The number of MCI cases in older migrants living in Europe and in each of the 32 considered countries was estimated by multiplying the number of migrants, provided by Eurostat, with the age-specific prevalence rates, derived by the harmonized data produced by the COSMIC collaboration and based on different operational definitions of MCI.

**Results:**

Nearly 686,000 cases of MCI were estimated in the extended EU by applying age-specific prevalence rates based on the International Working Group criteria. Higher figures were obtained when the Clinical Dementia Rating- and the Mini Mental State Examination-based criteria were applied. The proportion of MCI cases in migrant subjects ranged from 1.1% (Romania) to 54.1% (Liechtenstein) (median: 8.4%; IQR: 4.7%–14.2%).

**Conclusions:**

MCI represents and will increasingly constitute a relevant issue in the migrant population living in Europe. The present data reinforce the need of developing approaches and models of care that may be diversity-sensitive and inclusive for a culturally variegated population.

## INTRODUCTION

Mild cognitive impairment (MCI) is commonly intended as a decline in the individual’s cognitive performances not resulting in a significant reduction of functional independence and social or occupational functioning [1]. It is therefore conceived as an intermediate stage between normal cognition and dementia [2]. MCI is stimulating growing research and clinical interest. In fact, based on the available evidence, it represents a robust risk factor for future dementia [3]. At the same time, it is increasingly regarded as a promising phase for implementing dementia prevention strategies, as also indirectly suggested by the observed potential for clinical improvement/reversion to normal cognition [4]. As a proof, 349 randomized controlled trials testing novel pharmacological or non-pharmacological interventions targeting MCI are currently registered on the clinicaltrials.gov website (as of June 2019).

The detection of MCI is commonly triggered by a concern/complaint regarding a change in cognition from the subject, an informant, or a clinician. The clinical diagnosis then requires a standardized evaluation of the person’s cognitive functioning with the aim of providing the evidence of an objective impairment, in relation to normative parameters, in one or more cognitive domains [1]. Accordingly, cognitive testing has a central role in the so far adopted operational definitions of this condition [1, 5]. Moreover, the neuropsychological assessment is essential to properly characterize and classify this clinical construct (e.g., amnesic MCI versus non-amnesic MCI; single-domain MCI versus multi-domain MCI) and to more precisely stratify the individual risk profile in terms of conversion to dementia [6].

The identification and management of MCI may, however, be affected by diverse ethno-cultural determinants possibly influencing the personal and social perception and judgment of the one’s cognitive functioning as well as the reliability of objective cognitive assessment. These reflections may therefore assume a special relevance in migrants and subjects composing minority groups. In fact, it has been shown that these individuals may have different attitudes toward mental and cognitive disturbances [7, 8] and frequently have delayed contact with dedicated healthcare and social services [9]. Moreover, in Western countries, there is still a paucity and a scarce adoption of instruments and tools supporting a culture-sensitive (and, thus, more reliable) evaluation of cognitive performance [10, 11]. The considerations are gaining further relevance in light of the ongoing sociodemographic transitions consisting in the progressive aging not only of native populations but also of subjects with a migration background [12].

The aim of the present study is to provide an estimate of the number of MCI cases occurring in the migrant population living in the extended European Union (EU) in 2018 to start gaining awareness of this phenomenon.

## MATERIALS AND METHODS

### Older migrants in Europe

In the present study, “migrants” were operationally defined as those individuals living in a given European country but born abroad, regardless of the length of stay and the causes for the migration [13]. Data provided by the Statistical Office of the European Union, Eurostat (http://ec.europa.eu/eurostat/web/population-demography-migration-projections/population-data/database; database: “Population on 1 January by age group, sex and country of birth” [migr_pop3ctb]) were used to calculate the number of migrants, aged 60 years or older, living in Europe. Information was available for the 28 countries of the EU and the four countries composing the European Free Trade Association (i.e., Iceland, Liechtenstein, Norway, and Switzerland). All data were updated to August 2019 and referred to the subjects living in each country on 1 January 2018.

### Mild cognitive impairment prevalence rates

The age-specific prevalence rates of MCI were derived by the data provided by the Cohort Studies of Memory in an International Consortium (COSMIC) collaboration [14]. These estimates were calculated by applying different MCI classifications to the harmonized data coming from 11 longitudinal population-based studies on cognitive aging from USA, Europe, Asia, and Australia. Specifically, three commonly adopted operational definitions of MCI were applied to the participants aged 60 to 89 years recruited in these studies:

1) International Working Group criteria (IWG) [5]. MCI is identified by the presence of four criteria: absence of dementia (mostly ascertained with the DSM-IV criteria); no or minimal functional impairment (i.e., dependence in ≤two instrumental activities of daily living); subjective memory/cognitive complaints or concerns; and objective cognitive impairment (i.e., a score within the bottom 6.681%, or equivalently more than 1.5 SDs below the mean, of the scores for a given cognitive domain within the relevant study’s sample). MCI is further classified into amnesic (aMCI), when the memory domain is impaired, and non-amnesic (naMCI), when the impairment occurs in any of the other cognitive domains.
2) Mini-Mental State Examination (MMSE) score from 24 to 27 (inclusive) [15].
3) Clinical Dementia Rating (CDR) of 0.5 [16].

### Estimated cases of mild cognitive impairment among migrants in Europe

The number of MCI cases in migrants from 60 to 89 years old living in Europe and in the 32 considered countries was estimated by multiplying the number of migrants with the age-specific prevalence rates. For each nation, we also estimated the proportion of MCI cases occurring in migrants (calculated as the ratio between the estimated cases in migrants and in the overall population).

## RESULTS

A total of 12,730,960 migrants aged 60–89 years (women 55.1%) lived in Europe in 2018 (Table 1), with national estimates ranging from 3,326 in Iceland to 3,741,052 in Germany (Table 2).

**Table 1:** Estimated cases of mild cognitive impairment among migrant subjects living in the 32 considered countries based on different operational definitions

Definition of MCI	Prevalence (%)^*^	Migrants (*n* =)^#^	Estimated (*n* =)
**IWG criteria**
**MCI**
60–69 y	4.5	6,222,201	279,999
70–79 y	5.8	4,324,595	250,827
80–89 y	7.1	2,184,164	155,076
Total		12,730,960	685,902
**MCI subtypes**
aMCI (60–89 y)	2.0	12,730,960	254,619
naMCI (60–89 y)	3.9	12,730,960	496,507
**MMSE (60**–**89 y)**	12.0	12,730,960	1,527,715
**CDR (60**–**89 y)**	9.0	12,730,960	1,145,786

aMCI, amnestic mild cognitive impairment; naMCI, non-amnestic mild cognitive impairment; CDR, Clinical Dementia Rating; IWG, International Working Group; MCI, mild cognitive impairment; MMSE, Mini-Mental State Examination. ^*^Prevalence rates were taken from [14]. ^#^Source: Eurostat (http://ec.europa.eu/eurostat/web/population-demography-migration-projections/population-data/database). Data are updated to 2019 and refer to subjects living in each country on 1 January 2018.

**Table 2:** Estimated cases of mild cognitive impairment in migrant subjects living in each of the 32 considered countries based on different operational definitions

Country	Migrants (60–89 y)	Estimated MCI cases				
		IWG criteria			MMSE	CDR
		MCI	aMCI	naMCI
Belgium	361,766	19,033	7,235	14,109	43,412	32,559
Bulgaria	29,605	1,572	592	1,155	3,553	2,664
Czech Republic	44,670	2,214	893	1,742	5,360	4,020
Denmark	85,825	4,433	1,717	3,347	10,299	7,724
Germany	3,741,052	208,545	74,821	145,901	448,926	336,695
Estonia	101,099	5,521	2,022	3,943	12,132	9,099
Ireland	96,381	5,065	1,928	3,759	11,566	8,674
Greece	194,579	10,043	3,892	7,589	23,349	17,512
Spain	802,273	41,558	16,045	31,289	96,273	72,205
France	2,381,691	126,872	47,634	92,886	285,803	214,352
Croatia	196,836	10,544	3,937	7,677	23,620	17,715
Italy	685,228	34,655	13,705	26,724	82,227	61,671
Cyprus	21,923	1,109	438	855	2,631	1,973
Latvia	143,536	7,871	2,871	5,598	17,224	12,918
Lithuania	58,078	3,091	1,162	2,265	6,969	5,227
Luxembourg	46,003	2,392	920	1,794	5,520	4,140
Hungary	107,224	5,788	2,144	4,182	12,867	9,650
Malta	9,534	500	191	372	1,144	858
Netherlands	402,581	20,928	8,052	15,701	48,310	36,232
Austria	303,160	15,981	6,063	11,823	36,379	27,284
Poland	315,612	19,521	6,312	12,309	37,873	28,405
Portugal	132,550	6,847	2,651	5,169	15,906	11,930
Romania	48,184	2,703	964	1,879	5,782	4,337
Slovenia	70,889	3,654	1,418	2,765	8,507	6,380
Slovakia	63,177	3,376	1,264	2,464	7,581	5,686
Finland	31,475	1,597	630	1,228	3,777	2,833
Sweden	350,709	18,633	7,014	13,678	42,085	31,564
United Kingdom	1,351,311	72,275	27,026	52,701	162,157	121,618
Iceland	3,326	168	67	130	399	299
Liechtenstein	4,879	255	98	190	585	439
Norway	78,064	3,986	1,561	3,044	9,368	7,026
Switzerland	467,740	25,172	9,355	18,242	56,129	42,097

aMCI, amnestic mild cognitive impairment; naMCI, non-amnestic mild cognitive impairment; CDR, Clinical Dementia Rating; IWG, International Working Group; MCI, mild cognitive impairment; MMSE, Mini-Mental State Examination.

Nearly 686,000 cases of MCI were estimated in this population by applying age-specific prevalence rates based on the IWG criteria. Higher figures were obtained when the CDR- and the MMSE-based criteria were applied, increasing to almost 1.1 million and 1.5 million cases, respectively (Table 1).

As evident in Table 2, a relevant heterogeneity was observed at the national level, with IWG criteria-founded estimates widely ranging between 168 (Iceland) and 208,545 cases (Germany) (median: 5,655; IQR: 2,625–19,155). The ranges calculated by adopting the other classifications were: 299 to 336,695 cases (median: 9,375; IQR: 4,287–31,813) for the CDR-based definition; and 399 to 448,926 cases (median: 12,499; IQR: 5,717–42,417) when the MMSE score of 24–27 was considered.

**Fig.1:**
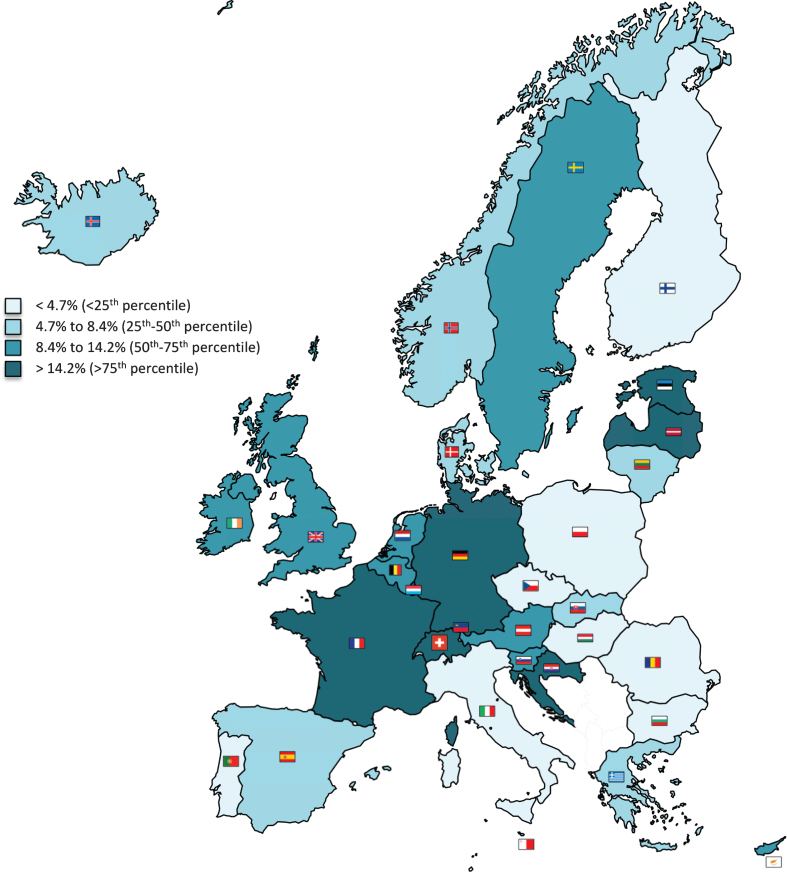
Proportion of mild cognitive impairment cases occurring in migrants in the 32 considered countries. Estimated cases of mild cognitive impairment in migrants and in the overall population were calculated by considering the International Working Group definition.

The proportion of MCI cases in migrant subjects (i.e., the ratio between the estimated cases in migrants and in the general population) ranged from 1.1% (Romania) to 54.1% (Liechtenstein) (median: 8.4%; IQR: 4.7%–14.2%) (Table 3 and Fig. 1).

**Table 3:** Proportion of mild cognitive impairment cases occurring in migrants in the 32 considered countries

Country	Estimated cases in migrants (n)^*^	Estimated cases in the overall population (n)^*^	Estimated % of cases in migrants
Belgium	19,033	147,579	12.9
Bulgaria	1,572	103,619	1.5
Czech Republic	2,214	142,235	1.6
Denmark	4,433	76,064	5.8
Germany	208,545	1,220,075	17.1
Estonia	5,521	17,990	30.7
Ireland	5,065	47,262	10.7
Greece	10,043	158,819	6.3
Spain	41,558	611,676	6.8
France	126,872	888,867	14.3
Croatia	10,544	59,225	17.8
Italy	34,655	916,865	3.8
Cyprus	1,109	9,623	11.5
Latvia	7,871	27,169	29.0
Lithuania	3,091	38,671	8.0
Luxembourg	2,392	6,159	38.8
Hungary	5,788	133,303	4.3
Malta	500	6,224	8.0
Netherlands	20,928	224,773	9.3
Austria	15,981	113,211	14.1
Poland	19,521	474,857	4.1
Portugal	6,847	151,911	4.5
Romania	2,703	255,103	1.1
Slovenia	3,654	28,522	12.8
Slovakia	3,376	62,070	5.4
Finland	1,597	80,442	2.0
Sweden	18,633	134,234	13.9
United Kingdom	72,275	821,397	8.8
Iceland	168	3,515	4.8
Liechtenstein	255	472	54.1
Norway	3,986	61,685	6.5
Switzerland	25,172	106,612	23.6

^*^Estimated cases of mild cognitive impairment in migrants and in the overall population were calculated by considering the International Working Group definition.

## DISCUSSION

To the best of our knowledge, the present study constitutes the first attempt to explore the magnitude of the issue of MCI occurring in migrants living in Europe. Our estimates, combined with those relating to dementia cases in the same population [17], suggest that more than one million migrants living in our continent is expected to be affected by a cognitive disorder, thus potentially referring to clinical and social facilities in the host countries. Accordingly, in several nations, a relevant proportion of cognitive disturbances is probably involving foreign-born individuals with important implications in terms of diagnostic accuracy, provision of care, and social support. These estimates are strongly influenced by the adopted operational definitions of MCI and are projected to markedly increase in the next future due to the current sociodemographic transformations [12]. In this regard, adopting the same analytic methods and considering the more stringent IWG criteria, it can be assumed that the number of MCI cases in migrants living in the extended EU has shown a 34% -increase in just 4 years, passing from 511,624 in 2014 to 685,902 in 2018.

Some potential limitations should be considered when interpreting these findings. First, the existence of possible differences in terms of MCI prevalence across diverse ethnic groups could not be neglected. We assumed that both natives and individuals migrating from different World regions shared the same risk of MCI, thus applying the same age-specific prevalence rates to the heterogeneous autochthonous and immigrant populations. Indeed, there is emerging evidence that cognitive disturbances may be more prevalent in specific groups and ethnicities. For instance, a study recruiting 2,254 participants aged 55 years or older in the Netherlands revealed that MCI was three times more frequent in most non-western migrants compared to the native Dutch population. In addition, a relevant variability of MCI prevalence across different immigrant groups was observed [18]. Such heterogeneity can be attributed to various determinants (e.g., vascular risk factors, educational level, lifestyles, physical activity, social interactions [19–21]) that have robustly been associated with the risk of cognitive disturbances and that were unavailable for the present analysis. The decision to base our analysis on the data produced by the COSMIC collaboration [14] (and not on other MCI prevalence data available in the literature) stems from the aim of providing different estimates of the phenomenon of interest according to alternative operationalizations of MCI that are widely adopted in the routine practice. For instance, the MMSE, despite being poorly sensitive and not recommended for the detection of MCI [22], is still largely used for its identification in clinical settings [23]. This approach has allowed us to frame the phenomenon taking into account the variability in its measurement. However, it should be noticed that the present estimates are not far from those that can be obtained from more recent MCI prevalence data. In fact, nearly 1,350,000 MCI cases can be estimated in the same European migrant population by applying the age-specific rates provided by the American Academy of Neurology (AAN) guideline on MCI, derived from studies targeting both MCI and related constructs (e.g., cognitive impairment no dementia (CIND)) [24]. Unfortunately, the adopted prevalence rates were available only for subjects aged less than 90 years. Therefore, we could not estimate the number of MCI cases in the oldest migrants, thus producing a possible underestimation of the phenomenon. The choice of not presenting our findings separately for men and women was instead supported by the fact that, in the pool analysis used as reference, MCI prevalence rates were substantially unaffected by sex [14].

In conclusions, MCI represents and will increasingly constitute a relevant issue in the migrant population living in Europe. This phenomenon remains to be characterized at the “real-world” level, thus merging the present epidemiological estimates with information coming from clinical and social services. Our data should inform clinicians, researchers, and policymakers on the need of developing approaches and models of care that may be diversity-sensitive and inclusive for a culturally variegated population. Given the centrality of the neuropsychological assessment in the detection of MCI, cross-cultural tools for the cognitive assessment should increasingly be used. In parallel, the possible role and involvement of professionals like interpreters and cultural mediators in the field of cognitive disturbances should be considered. Moreover, a greater effort should be made in order to understand the migrants’ attitudes, beliefs, and perceptions toward cognition and cognitive disorders.
